# Editorial: Methods & applications in theoretical and philosophical psychology: research methods in the study of consciousness

**DOI:** 10.3389/fpsyg.2024.1357788

**Published:** 2024-01-16

**Authors:** Gillie Gabay, Douglas A. MacDonald, Melanie Gillespie Rosen

**Affiliations:** ^1^Department of Social Sciences, Achva Academic College, Arugot, Israel; ^2^Department of Psychology, University of Detroit Mercy, Detroit, MI, United States; ^3^Department of Philosophy, Trent University, Peterborough, ON, Canada

**Keywords:** theories, editorial, methods, consciousness, applications

This editorial has been written as the endpoint of a successful Research Topic focusing on research methods in the study of consciousness. Scientific and philosophical interest in consciousness has burgeoned in recent years as have the methods used to study it. Our primary impetus in proposing the topic was to provide a forum to showcase some of the ever-growing variety of methods. Our hope is that doing so will help foster critical discussions regarding how these methods may contribute to the interdisciplinary development of knowledge of the human mind.

The five articles that comprise the Research Topic were written by scholars from nine countries (i.e., Argentina, Australia, Belgium, Canada, France, Germany, Netherlands, UK, and US) and embody a range of contemporary research foci and methods. Walter and Hinterberger discuss the notion of self-organized criticality (SOC), which is a way of improving system performance through the ability of a complex systems to undergo a second-order phase transition. They consider the possibility of SOC as a core property of the nervous system which holds the potential to offer a neurodynamic framework for understanding consciousness. To to assess this view, they performed a review of the available literature on SOC and discuss the array of applied methodologies employed in empirical research. They found that SOC indeed aligns with modern influential theories of consciousness.

Nayak and Griffiths completed an investigation about psychedelic experiences. Using traditional survey methods, they aimed at exploring how these experiences influence people's beliefs about consciousness and, in particular, their attributions of consciousness to different objects and life forms. Their findings suggest that psychedelics induce powerful alterations of consciousness that do impact how people understand the world around them, but in ways that may appear counter-intuitive. Individuals increase their attribution of consciousness to animals and inanimate objects, and surprisingly, these effects remained for extended periods of time, showing the effect in a survey taken years after the experience.

Alcaraz-Sánchez et al. analyzed minimal forms of awareness during sleep using micro-phenomenological interview, a technique that allows subjects to better focus on their experiences, along with several forms of qualitative analysis (e.g., phenomenological analysis, grounded theory, thematic analysis). The aim of their study was to identify the characteristics of objectless awareness during sleep. They identified an experiential phase of sleep simply called the “nothingness phase” that has unique features. This phase significantly reduced sense of self and physical embodiment, unfocused attention and involved an absence of visual imagery. The nothingness phase can be described as conscious awareness that lacks any content to the experience.

Orianne and Eustache undertook a detailed analysis of the concept of collective memory. Their article focused on the history of collective memory and described a current conflation with other memory concepts such as shared, collaborative, and social memory. The authors argued that collective memory, unlike social memory, is not a type of memory of a collective group. Instead, collective memory concerns a system of consciousness at the individual level which can be linked to social memory through communications with social groups and other social interactions.

Last, Trakas offered a critical discussion of a spatial model of memory called mental time travel (MMT). MTT has come to be applied to the conscious experience of remembering in that memory allows us to travel back in time. Drawing from the philosophical literature on time travel, Trakas tackles issues of both the ontology and phenomenal experience of MTT. A main goal of this article is to shed light on the distinct features that MMT in relation to memory. She draws on philosophical theories of time to elucidate the concept of MTT and evaluate its use a metaphor for remembering.

One novel and valuable feature of this research topic is the diversity of nationalities, research foci, and methods reflected in these papers. This research makes apparent that scientific inquiry into consciousness is still very much in its nascent stages while at the same time drawing attention to the potential of cross-cultural and cross-disciplinary collaboration. The editors of this research topic are of the conviction that a consilience of knowledge on consciousness could be, and should be, facilitated through the integration of theories and methods that have traditionally been kept separate and embraced by investigators from different disciplines. One currently available approach to inquiry that seems to offer such integration is neurophenomenology. This field of research reflects attempts to bring together the study of experience, cognition, and culture by interweaving phenomenology and neuroscience methods (see Laughlin and Rock, 2013 for an excellent overview of the histor and cognitive and cultural forms of this exciting approach). Although we are confident that readers will find each of the articles in this research topic interesting in its own right, we hope that this article collection will inspire scholars to seek out and develop new integrative methods.

## Author contributions

GG: Project administration, Writing – original draft. DM: Project administration, Writing – review & editing. MR: Project administration, Writing – review & editing.

## References

[B1] Laughlin C. D. Rock A. J. (2013). “Neurophenomenology: enhancing the experimental and cross-cultural study of brain and experience,” in The Wiley-Blackwell Handbook of Transpersonal Psychology, eds. H. L. Friedman, and G. Hartelius. Hoboken: John Wiley and Sons, 261–280.

